# Effective long‐term prophylaxis with lanadelumab in adolescents with hereditary angioedema: EMPOWER/ENABLE

**DOI:** 10.1111/pai.70072

**Published:** 2025-04-02

**Authors:** Raffi Tachdjian, Aleena Banerji, Paula J. Busse, Nancy Agmon‐Levin, John Anderson, Mauro Cancian, Giuseppe Spadaro, Carmen Enciu, Daniel Nova Estepan, Natalie Khutoryansky, Siddharth Jain, Andreas Recke

**Affiliations:** ^1^ Division of Allergy, Immunology and Rheumatology UCLA School of Medicine Los Angeles California USA; ^2^ Providence St. John Medical Center Santa Monica California USA; ^3^ Division of Rheumatology, Allergy and Immunology, Department of Medicine Massachusetts General Hospital, Harvard Medical School Boston Massachusetts USA; ^4^ Division of Allergy and Clinical Immunology Icahn School of Medicine at Mount Sinai New York New York USA; ^5^ Clinical Immunology, Angioedema and Allergy Institute The Center for Autoimmune Diseases, Sheba Medical Center, Tel HaShomer Hospital Ramat‐Gan Israel; ^6^ AllerVie Health Birmingham Alabama USA; ^7^ Department of Systems Medicine University Hospital of Padua Padua Italy; ^8^ Department of Translational Medical Sciences University of Naples Federico II Naples Italy; ^9^ Center for Basic and Clinical Immunology Research (CISI), WAO Center of Excellence University of Naples Federico II Naples Italy; ^10^ Takeda Development Center Americas, Inc. Lexington Massachusetts USA; ^11^ Department of Dermatology, Allergology and Venereology University of Lübeck Lübeck Germany

**Keywords:** adolescents, effectiveness, hereditary angioedema, lanadelumab, prophylaxis, safety

## Abstract

**Background:**

Symptoms of hereditary angioedema (HAE) typically first present during childhood, but the frequency/severity of attacks often increases at puberty. Real‐world data on long‐term HAE prophylaxis in adolescents are limited. We report pooled data from adolescent patients enrolled in two Phase 4 studies (EMPOWER, ENABLE) evaluating the effectiveness/safety of lanadelumab (monoclonal antibody directed against plasma kallikrein) for the prevention of HAE attacks.

**Methods:**

Adolescent patients (aged 12 to <18 years) with HAE‐C1INH enrolled in EMPOWER and ENABLE received open‐label lanadelumab 300 mg once every 2 weeks. Effectiveness outcomes were based on patient‐reported assessments of on‐treatment HAE attacks. Safety was assessed through the recording of treatment‐emergent adverse events (TEAEs) and serious adverse events. This analysis categorized patients as “new” or “established” lanadelumab patients.

**Results:**

Thirteen new and seven established patients on lanadelumab were included. The observed monthly attack rate in new patients fell from 3.8 (mean) and 2.8 (median) during the pre‐enrollment period to 0.65 (mean) and 0.21 (median) during the cumulative study period after lanadelumab initiation (84.2% and 92.9% reductions, respectively). In established patients, mean (SD) HAE attack rate (as treated) during the overall study period was 0.04 (0.03) attacks/month. Most HAE attacks were of mild/moderate severity. Nine new patients reported 42 TEAEs, mostly mild/moderate in severity, with 3 TEAEs reported as serious. Seven established patients reported 12 TEAEs (all mild/moderate and non‐serious). No TEAEs were related to lanadelumab.

**Conclusion:**

These data support lanadelumab's effectiveness/safety in adolescents with HAE, consistent with results from Phase 3 lanadelumab studies in mixed adult/adolescent populations.

**Clinical Trial Identifiers:**

NCT03845400 (EMPOWER) and NCT04130191 (ENABLE).

**Key message**

- There is currently a shortage of data on long‐term prophylaxis outcomes in adolescents with hereditary angioedema (HAE).
- In this study, we employed pooled data from 2 real‐world observational studies to assess the effectiveness and safety of lanadelumab in preventing HAE attacks in patients aged 12–17 years with HAE‐C1INH.
- Consistent with findings reported in mixed adult/adolescent populations, the study suggests that lanadelumab provides effective, safe, and tolerable long‐term prophylaxis against HAE attacks among adolescent patients in clinical practice.

## 1 INTRODUCTION

Hereditary angioedema (HAE) is a rare genetic disorder that manifests as spontaneous episodes of cutaneous or submucosal edema that affect the skin, abdomen, and upper airway.^1^, ^2^ The most common types of HAE are characterized by reduced plasma levels of C1 inhibitor (C1INH; HAE‐C1INH‐Type1) or dysfunctional C1INH activity (HAE‐C1INH‐Type2) due to mutations of the *SERPING1* gene.^1^, ^3^ The deficiency of C1INH leads to dysregulation of plasma kallikrein activity, excess bradykinin production, and activation of bradykinin B2 receptors, which in turn causes a localized increase in vascular permeability and tissue swelling.^1^, ^3^

HAE symptoms usually present during childhood, with 50% of patients experiencing their first attack before 10 years of age, and the majority of cases presenting by 20 years of age.^4^, ^5^, ^6^, ^7^ Although symptoms typically first present during childhood, the frequency and severity of HAE attacks often increase around puberty,^8^ thus worsening the burden of disease. The clinical symptoms of HAE can have a profound impact on patients' lives.^9^ HAE attacks that affect the skin manifest as swellings in the face, hands, arms, feet, and legs and can be disfiguring, painful, and debilitating.^10^, ^11^, ^12^ Abdominal HAE attacks affect the stomach and intestines, and clinical symptoms may include diffuse pain, vomiting, diarrhea, and abdominal cramping.^3^ Although laryngeal swelling is not the most common clinical presentation of HAE, it has the potential to be life‐threatening because of the risk of asphyxiation; approximately 50% of patients will experience ≥1 of these episodes.^3^, ^13^ The clinical symptoms and unpredictability of HAE attacks can exact a substantial physical, psychological, and social burden on patients. Patients with untreated or inadequately controlled disease may experience significant disruptions in their work, school, career choices, and social activities.^14^

Management strategies for HAE attacks include on‐demand treatment, short‐term prophylaxis, where a patient‐specific angioedema‐inducing situation is anticipated, including a surgical or invasive medical procedure, and long‐term prophylaxis (LTP).^1^, ^15^ Effective LTP has the potential to normalize a patient's life, which is an important treatment goal.^1^ There are several options for LTP in patients aged ≥12 years, including plasma‐derived C1INH concentrates and plasma kallikrein inhibitors, such as lanadelumab and berotralstat, which are recommended as first‐line agents for LTP of HAE.^1^ In patients with HAE aged <12 years, treatment options were previously limited, with C1INH and icatibant as the only on‐demand treatments approved in this patient population.^1^ Plasma‐derived C1INH is the recommended first‐line short‐term prophylaxis in children with HAE.^1^ LTP options for children were, until recently, limited to plasma‐derived C1INH, with antifibrinolytics preferred over androgens when C1INH is not available.^1^

Lanadelumab is a fully human monoclonal antibody that reduces bradykinin production through inhibition of active plasma kallikrein proteolytic activity.^16^, ^17^ Lanadelumab is approved for the prophylaxis of HAE attacks in patients aged ≥2 years in the United States^18^ and Europe^19^ and for patients aged ≥12 years in many other countries.^20^ Transitioning patient care between childhood and adulthood is facilitated by the possibility of using flexible dosing with lanadelumab.^8^ LTP with lanadelumab in pediatric and adolescent patients offers the advantage of fewer injections than needed for subcutaneously administered C1INH and a reduced dosing frequency than required for oral medication, such as berotralstat.

Real‐world data are invaluable to gain further information on the effectiveness and safety of therapies in clinical practice settings and to complement the outcomes from controlled clinical trials. EMPOWER (NCT03845400), a Phase 4 study that was conducted at multiple sites across the United States and Canada, aimed to evaluate the real‐world effectiveness of lanadelumab, as measured by HAE attack rates before and after lanadelumab initiation. Similarly, ENABLE (NCT04130191) a Phase 4 study conducted in several countries in Europe, as well as Israel and Kuwait, sought to evaluate the effectiveness of lanadelumab in preventing the occurrence of HAE attacks in real‐world clinical practice.

Because EMPOWER and ENABLE are observational, non‐interventional studies, lanadelumab treatment was administered in accordance with the country‐approved product labeling at the time of study entry, and both studies included patients aged ≥12 years. The onset of HAE symptoms during early childhood may predict a more severe disease course, including increased frequency of attacks.^21^ Also, studies of children with HAE from Israel and Hungary show impaired quality of life and increased anxiety compared with healthy controls; therefore, adequate protection against HAE attacks with an effective LTP option is imperative.^22^, ^23^ Given the paucity of data on LTP outcomes in adolescents with HAE, this current post hoc analysis assessed lanadelumab treatment outcomes in adolescents (aged 12–17 years) with HAE using pooled data from the EMPOWER final data and the third interim analysis of ENABLE.

## 2 METHODS

### 2.1 Study population

Patients diagnosed with HAE who were receiving care from a physician were enrolled in the EMPOWER and ENABLE studies. Patients or their legal representatives provided informed consent to participate in the studies, and patients/caregivers had to be able to use a smartphone for study data collection. The availability of information on the number, nature, and treatment of HAE attacks for the 3 months before enrollment was a specified inclusion criterion for ENABLE. In EMPOWER, patient's recall or physician assessment of the patient's medical history up to, but not exceeding, 6 months before enrollment was sufficient. Patients participating in an interventional clinical trial at the time of enrollment or considered unsuitable by the treating physician/investigator were excluded.

### 2.2 Study design

EMPOWER and ENABLE are non‐interventional, prospective, multicenter Phase 4 studies in patients with HAE treated with lanadelumab according to current product labeling in real‐world clinical practice. In ENABLE, all patients who initiated lanadelumab during the eligibility period and met the other inclusion/exclusion criteria were included in the study. In EMPOWER, patients who were either new to lanadelumab or established on lanadelumab and met the other inclusion/exclusion criteria were included in the study. New lanadelumab patients were defined as patients who had not initiated lanadelumab at the time of enrollment or who had initiated lanadelumab before enrollment but had received <4 doses before the enrollment date (i.e., had not attained steady‐state levels of lanadelumab). Established lanadelumab patients were patients who initiated lanadelumab before enrollment and had received ≥4 doses, with the most recent dose administered <70 days before enrollment.

For both studies, the observation and data collection period extended for up to 36 months post enrollment. Patients completed an attack diary using a smartphone application to self‐report HAE attacks at the time of occurrence and capture any on‐demand therapies administered to treat these attacks (predominantly plasma‐derived C1INH and icatibant). Data were reported at study start and every month for the first 3 months, and then every 6 months thereafter until study end in ENABLE and at study start and every 6 months thereafter until study end in EMPOWER. For both studies, comprehensive data collected by the treating physician during routine patient visits were entered into electronic case report forms (eCRFs) at enrollment and every 6 months thereafter until study end.

EMPOWER and ENABLE were conducted in accordance with International Conference on Harmonization of Good Clinical Practice guidelines and the principles of the Declaration of Helsinki, as well as other applicable local ethical and regulatory requirements. The study protocols were approved by independent institutional review boards as well as by site‐specific independent ethics committees, as applicable by regional regulations.

### 2.3 Study assessments

#### 2.3.1 HAE attacks

Patient‐reported assessments of on‐treatment HAE attacks included the number, severity, anatomical location, and duration of HAE attacks, as well as on‐demand treatment usage. Attacks were reported as mild (defined as transient or mild discomfort, with no medical intervention/therapy required), moderate (defined as mild to moderate limitation in activity, with some assistance possibly needed; no or minimal medical intervention/therapy required), severe (defined as marked limitation in activity, with some assistance usually required; medical intervention/therapy required, with hospitalization possible), or life‐threatening (defined as extreme limitation in activity, significant assistance required; significant medical intervention/therapy required, with hospitalization or hospice care probable).

#### 2.3.2 Safety

Safety was assessed through the recording of treatment‐emergent adverse events (TEAEs) and serious adverse events collected in the eCRF. Physician‐confirmed HAE attacks were also recorded as adverse events.

### 2.4 Statistical analyses

Summary statistics are reported for this post hoc analysis of adolescent patients enrolled in either ENABLE or EMPOWER. The data used in this analysis were pooled from a third interim analysis of ENABLE (study period for interim analysis 3 was December 11, 2019, to April 17, 2023) and EMPOWER (study period for final data was March 30, 2019, to October 11, 2022). Numerical variables were summarized using descriptive statistics. The full analysis set consisted of all patients in the safety set who had ≥1 post‐enrollment effectiveness outcome assessment. To assess the effectiveness of lanadelumab in patients newly initiated to lanadelumab, the mean (standard deviation [SD]) number of on‐treatment HAE attacks, the estimated mean attack rate (attacks per month), and 95% confidence intervals (CIs) during pre‐lanadelumab treatment, early state (time period from first dose of lanadelumab to Day 69), steady state (Day 70 onward), and cumulatively for early and steady states combined were calculated. Unadjusted (observation period as the only independent variable) and adjusted (sex and age at lanadelumab initiation as additional independent variables) incidence rate ratios (IRRs) were calculated relative to pre‐enrollment using a generalized linear model with negative binomial distribution and a log link. Percentage reductions in attack rate and IRR and associated 95% CI values were reported for the early state and steady‐state periods separately and cumulatively. To assess the effectiveness of lanadelumab among established patients, the HAE attack rate (as treated) during the overall study period was calculated. Statistical analyses were conducted using SAS^®^ version 9.4 (SAS Institute, Cary, NC, USA).

## 3 RESULTS

### 3.1 Patient population

Overall, 13 adolescent patients who were new to lanadelumab and 7 adolescent patients with established lanadelumab use were included in this analysis. Baseline demographics and clinical characteristics of patients are summarized in Table 1. Mean (SD) age of new lanadelumab patients was 15.2 (2.0) years, 53.8% were female, all were White, and weighed 38–144 kg (range). Ten patients had HAE‐C1INH‐Type1, 2 had HAE‐C1INH‐Type2, and 1 patient had undifferentiated (i.e., unspecified) HAE at the time of the interim analysis, which was later confirmed by the investigator to be HAE with normal C1INH (Table 1). Mean (SD) age of established lanadelumab patients was 15.7 (1.4) years, most were female (71.4%), all were White, and weighed 52–136 kg (range). Six patients had HAE‐C1INH‐Type1 and 1 had HAE‐C1INH‐Type2 (Table 1). Overall, 4 patients received LTP prior to enrollment and starting treatment with lanadelumab. Three of these patients received intravenous plasma‐derived C1INH for at least 1 year and up to 3 years prior to lanadelumab treatment. Another patient received subcutaneous plasma‐derived C1INH twice weekly for over 3.5 years prior to enrollment.

**TABLE 1:** Baseline demographics and clinical characteristics of adolescent patients with HAE (FAS).

	New to lanadelumab (*N* = 13)	Established on lanadelumab (*N* = 7)
Age (years)		
Mean (SD)	15.2 (2.0)	15.7 (1.4)
Range	12–17	14–17
Female, *n* (%)	7 (53.8)	5 (71.4)
White, *n* (%)	13 (100)	7 (100)
Height (cm)		
*n*	11	7
Mean (SD)	168.5 (9.8)	171.9 (6.5)
Median (range)	170.0 (145–180)	170.0 (166–181)
Weight (kg)		
*n*	11	7
Mean (SD)	77.4 (32.3)	87.8 (34.8)
Median (range)	63.5 (38–144)	75.1 (52–136)
HAE‐C1INH type, *n* (%)	13	7
Type1	10 (76.9)	6 (85.7)
Type2	2 (15.4)	1 (14.3)
HAE‐nC1INH	1 (7.7)	0

Abbreviations: C1INH, C1 inhibitor; FAS, full analysis set; HAE, hereditary angioedema; nC1INH, normal C1 inhibitor; SD, standard deviation.

For adolescent patients in EMPOWER and ENABLE, the mean ± SD duration of lanadelumab treatment was 680.5 ± 291.5 days in new lanadelumab patients and 947.7 ± 87.0 days in established patients. Throughout the study period, 5 of the 20 adolescent patients had lanadelumab dose intervals extended from every 2 weeks (Q2W) dosing periods. Three of these patients had intervals extended to every 4 weeks (Q4W), 1 extended to Q4W dosing after interim every 3 weeks (Q3W) dosing, and another extended to Q3W dosing and maintained this regimen until the end of the study.

### 3.2 Effectiveness of lanadelumab

**FIGURE 1:**
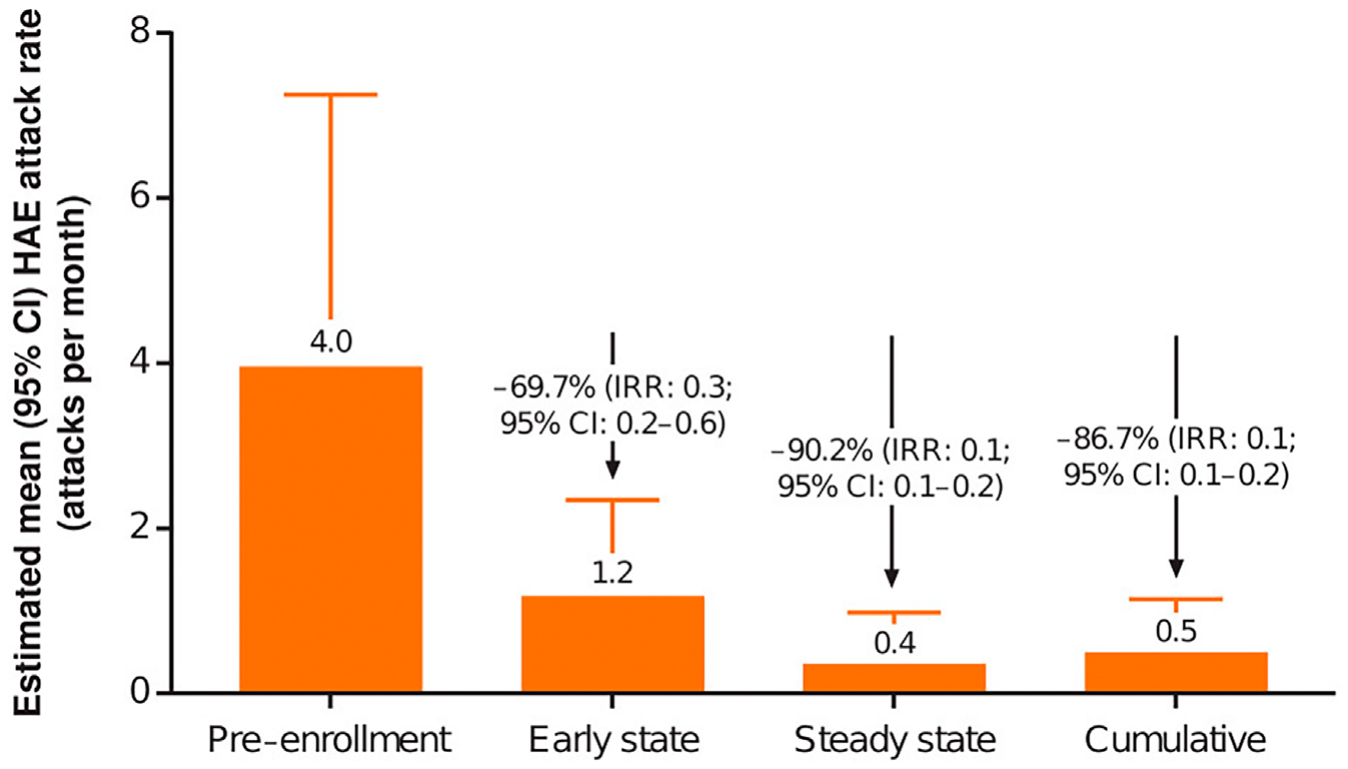
Model‐estimated monthly HAE attack rates and IRR relative to pre‐lanadelumab for new patients for each lanadelumab observation period (FAS). CI, confidence interval; FAS, full analysis set; HAE, hereditary angioedema; IRR, incidence rate ratio.

**FIGURE 2:**
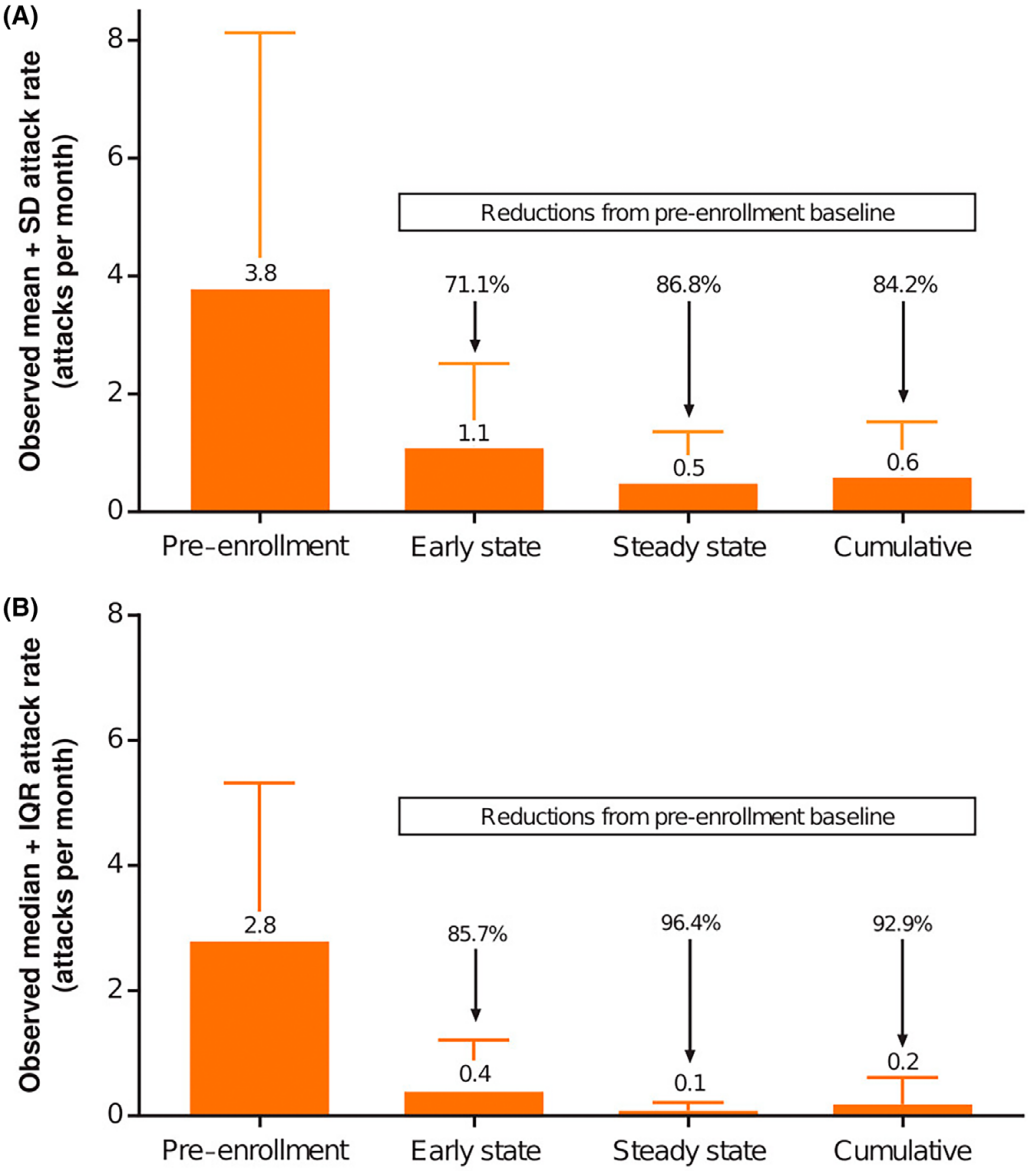
(A) Observed (mean + SD) monthly HAE attack rates among new patients for each lanadelumab observation period (FAS). (B) Observed (median + IQR) monthly HAE attack rates among new patients for each lanadelumab observation period (FAS). FAS, full analysis set; HAE, hereditary angioedema; IQR, interquartile range; SD, standard deviation.

For new lanadelumab patients, the mean (SD) number of attacks (over mean [SD] patient duration in time period) was 13.8 (14.3) attacks (152.5 [118.6] days) at pre‐enrollment, 2.8 (3.5) attacks (69.4 [0.5] days) at early state, 7.2 (12.6) attacks (611.2 [291.4] days) at steady state, and 10.0 (14.8) attacks (680.5 [291.5] days) cumulatively. For established patients, a mean (SD) of 1.3 (1.1) attacks was reported during the overall study period (mean [SD], 947.7 [87.0] days). Adjusted model‐estimated mean attack rate (attacks/month) and IRR for each lanadelumab observation period are shown in Figure 1 for new lanadelumab patients. The adjusted mean monthly HAE attack rate in new patients decreased by 86.7% post lanadelumab, from 3.99 (95% CI, 2.19–7.25) at baseline (ENABLE, composite pre lanadelumab; EMPOWER, pre‐enrollment) to 0.53 (95% CI, 0.25–1.14) during the cumulative period post lanadelumab (IRR, 0.13, 95% CI, 0.08–0.24). The observed monthly attack rate likewise fell from 3.8 (mean) and 2.8 (median) during the pre‐enrollment period to 0.65 (mean) and 0.21 (median) during the cumulative period, representing reductions of 84.2% and 92.9%, respectively (Figure 2). In established patients, the mean (SD) HAE attack rate (as treated) during the overall study period was 0.04 (0.03) attacks/month.

### 3.3 HAE attack characteristics

**FIGURE 3:**
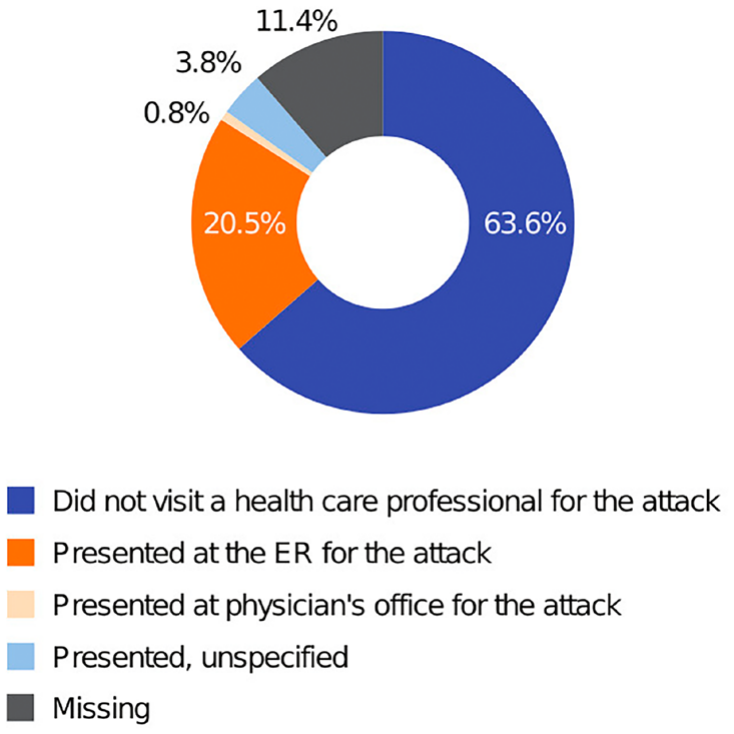
Health care encounters for HAE attacks in new patients (FAS). Information on health care encounters among established patients is not available. ER, emergency room; HAE, hereditary angioedema; FAS, full analysis set.

HAE attack characteristics are summarized in Table 2 and Figure 3. New lanadelumab patients experienced a total of 132 HAE attacks during the study, and for the majority of these attacks (63.6%), patients reported that they did not visit a health care professional. For the 33 attacks (25.0%) where health care professional assistance was reported to have been sought, 27 resulted in emergency room encounters, which were all confined to 3 new lanadelumab patients. A total of 125 of the attacks were treated, most commonly with icatibant or plasma‐derived C1INH. Most HAE attacks were classified as moderate in severity. Data on attack location were missing for most of the attacks, but for those patients who did report location, attacks predominantly affected the abdomen or periphery.

**TABLE 2:** HAE attack characteristics (FAS).

	New to lanadelumab (*N* = 13)	Established on lanadelumab (*N* = 7)
Number of HAE attacks	132	9
HAE treatment for the attack, *n* (%)		
*n*	132	9
Yes	125 (94.7)	6 (66.7)
No	7 (5.3)	3 (33.3)
HAE treatment received, *n* (%)		
*n*	125	6
Plasma‐derived C1INH	41 (32.8)	0
Recombinant C1INH	0	2 (33.3)
Icatibant	51 (40.8)	3 (50.0)
Other	9 (7.2)	1 (16.7)
Missing	24 (19.2)	0
Severity of HAE attack, *n* (%)		
*n*	132	9
Grade 1 (mild)	17 (12.9)	2 (22.2)
Grade 2 (moderate)	86 (65.2)	3 (33.3)
Grade 3 (severe)	29 (22.0)	4 (44.4)
Body part affected by HAE attack, *n* (%)		
*n*	132	9
Peripheral	7 (5.3)	4 (44.4)
Airways	1 (0.8)	1 (11.1)
Abdomen	13 (9.8)	3 (33.3)
Testicle	2 (1.5)	0
Head	0	1 (11.1)
Missing	109 (82.6)	0

Abbreviations: C1INH, C1 inhibitor; FAS, full analysis set; HAE, hereditary angioedema.

Established lanadelumab patients experienced 9 HAE attacks, with most attacks affecting either the abdomen or periphery. Data on health care encounters were missing for these established lanadelumab patients. Six attacks were treated, mainly with either recombinant C1INH or icatibant. HAE attack intensity was reported as mild (22.2%), moderate (33.3%), or severe (44.4%).

### 3.4 Safety

No lanadelumab‐related TEAEs or injection site reactions were reported for new or established patients. Nine new lanadelumab patients reported a total of 42 unrelated TEAEs, 20 (47.6%) of which were mild, 17 (40.5%) moderate, and 5 (11.9%) severe in intensity (Table 3). Most of the TEAEs were not considered serious (*n* = 39; 92.9%). Seven established lanadelumab patients reported 12 unrelated TEAEs, of which 9 (75.0%) were mild and 3 (25.0%) were moderate in severity. None of these TEAEs were considered serious. No patients discontinued lanadelumab treatment due to TEAEs, and there were no deaths among either new or established lanadelumab patients in either study. No TEAE was reported in the sole study patient weighing <40 kg.

**TABLE 3:** TEAEs (excluding HAE attack adverse events) during lanadelumab long‐term prophylaxis (FAS).

	New to lanadelumab (*N* = 13)		Established on lanadelumab (*N* = 7)	
	Patients, *n*/*N* (%)	Events, *n*/*N* (%)	Patients, *n*/*N* (%)	Events, *n*/*N* (%)
Any TEAE	9/13 (69.2)	42/42	7/7 (100)	12/12 (100)
COVID‐19 infection	3/9 (33.3)	3/42 (7.1)	5/7 (71.4)	6/12 (50.0)
Hyperuricemia	2/9 (22.2)	2/42 (4.8)	–	–
Toothache/dental pulpitis	2/9 (22.2)	7/42 (16.7)	–	–
Abdominal pain	1/9 (11.1)	1/42 (2.4)	–	–
Alopecia	1/9 (11.1)	1/42 (2.4)	–	–
Angioedema	1/9 (11.1)	1/42 (2.4)	–	–
Anxiety	–	–	1/7 (14.3)	1/12 (8.3)
Arthralgia	1/9 (11.1)	1/42 (2.4)	–	–
Asthma	–	–	1/7 (14.3)	1/12 (8.3)
Bell's palsy	1/9 (11.1)	1/42 (2.4)	–	–
Cold urticaria	1/9 (11.1)	1/42 (2.4)	–	–
Depression	1/9 (11.1)	1/42 (2.4)	1/7 (14.3)	1/12 (8.3)
Device expulsion	1/9 (11.1)	1/42 (2.4)	–	–
Endometriosis	1/9 (11.1)	1/42 (2.4)	–	–
Fracture (ankle)	–	–	1/7 (14.3)	1/12 (8.3)
Foreign body	1/9 (11.1)	1/42 (2.4)	–	–
Hemiparesis	1/9 (11.1)	3/42 (7.1)	–	–
Hemorrhagic diathesis	1/9 (11.1)	1/42 (2.4)	–	–
Hypertension	1/9 (11.1)	1/42 (2.4)	–	–
Hypoesthesia	1/9 (11.1)	1/42 (2.4)	–	–
Ingrowing nail	1/9 (11.1)	1/42 (2.4)	–	–
Iron deficiency	1/9 (11.1)	1/42 (2.4)	–	–
Joint hyperextension	1/9 (11.1)	1/42 (2.4)	–	–
Knee impingement syndrome	–	–	1/7 (14.3)	1/12 (8.3)
Limb injury	1/9 (11.1)	1/42 (2.4)	–	–
Lymphadenopathy	1/9 (11.1)	1/42 (2.4)	–	–
Migraine	1/9 (11.1)	1/42 (2.4)	–	–
Nausea	1/9 (11.1)	1/42 (2.4)	–	–
Oropharyngeal pain	1/9 (11.1)	1/42 (2.4)	–	–
Peripheral swelling	1/9 (11.1)	5/42 (11.9)	–	–
Type 2 diabetes	1/9 (11.1)	1/42 (2.4)	–	–
Wheezing	–	–	1/7 (14.3)	1/12 (8.3)

Abbreviations: COVID‐19, coronavirus disease 2019; FAS, full analysis set; HAE, hereditary angioedema; TEAE, treatment‐emergent adverse event.

## 4 DISCUSSION

Recruitment of adolescent patients into clinical trials is often hampered by limited patient numbers and strict eligibility criteria, highlighting why data obtained in the real‐world setting following drug approval are essential for informing treating physicians.

This ad hoc analysis of pooled real‐world data from the EMPOWER and ENABLE studies suggests that adolescent patients, who comprised a small minority (approximately 6% and 11%, respectively) of the lanadelumab‐treated populations of these 2 studies, experienced similar HAE attack frequencies to adult patients in these studies.^24^ The observed baseline (pre‐lanadelumab) mean and median attack rates of 3.8 and 2.8 attacks/month, respectively, among adolescent patients new to lanadelumab were similar to the pre‐lanadelumab mean and median rates for new patients of 3.8 and 2.8 attacks per month, respectively, in ENABLE (Takeda unpublished data) and were higher than the pre‐enrollment (up to 6 months prior to enrollment) mean and median attack rates of 1.6 and 0.5 attacks per month, respectively, among new patients in EMPOWER (Takeda unpublished data). For the pooled adolescent subgroup, an early state mean and median reduction in observed monthly HAE attack rate of 71.1% and 85.7%, respectively, was reported after initiating lanadelumab prophylaxis in this real‐world clinical setting. The effectiveness of lanadelumab was sustained over time, as shown by a mean and median reduction in monthly attack rate of 86.8% and 96.4%, respectively, at steady state compared with the pre‐lanadelumab period, and an overall mean and median reduction of 84.2% and 92.9%, respectively. These reductions in HAE attack rate are consistent with findings from a mixed adult and adolescent population and adolescent‐only population in the open‐label extension of the Phase 3 HELP trial (HELP OLE) and the mixed adult and adolescent population from the EMPOWER study. In HELP OLE, lanadelumab prophylaxis reduced the mean monthly HAE attack rate by 87.4% compared with baseline in the mixed adolescent and adult patient population,^25^ whereas in the subpopulation of 21 adolescent patients (aged 12–18 years) enrolled in HELP OLE, the mean monthly HAE attack rate was reduced by 94.7%.^26^

For established lanadelumab patients among the pooled adolescent subgroup, mean (SD) and median attack rates of 0.04 (0.03) and 0.0 attacks/month, respectively, were observed for the overall study period. This finding aligns with previously published data from the HELP study demonstrating the rapid and sustained prevention of HAE attacks with lanadelumab.^27^ Additionally, while attack rates were not analyzed on a monthly basis after Day 70, a recent retrospective study in Europe evaluated the monthly attack‐free rate during lanadelumab treatment for up to 43 months.^28^ This study in 198 patients (including 4 adolescents) reported monthly attack‐free rates ranging from 82.7% to over 95% following lanadelumab treatment (compared with varying rates from 16.2% to 28.3% pre‐lanadelumab) reinforcing the long‐term sustained effectiveness that can be achieved with lanadelumab.

Several HAE attacks experienced by adolescent patients new to lanadelumab or established on lanadelumab were not treated with on‐demand therapy. While the majority of attacks experienced during lanadelumab treatment tended to be mild or moderate in severity, we agree with the WAO/EAACI recommendation that all attacks should be considered for on‐demand treatment and that attacks are treated as early as possible.^1^ A lack of on‐demand treatment is not a unique finding, as other studies have reported similar results.^29^, ^30^ Patients may opt not to treat their attacks for a number of reasons, including financial constraints that cause patients to save medications for more severe attacks, medication constraints caused by a lack of on‐hand treatment, or the perception that an attack is mild and will pass on its own. Given there is no finite treatment period for the clinical use of long‐term prophylactic agents in the management of HAE, an understanding of the long‐term safety in adolescents is paramount. The TEAEs reported by the adolescent patients in EMPOWER and ENABLE were mostly mild or moderate in severity, and none were considered related to lanadelumab treatment. There were no reports of injection site reactions in adolescents participating in ENABLE or EMPOWER, while a total of 41.7% (35/84) and 42.5% (90/212) of patients participating in the HELP and HELP OLE studies, respectively, reported treatment‐related injection site reactions.^20^, ^25^ No adolescent patients discontinued treatment. These data support the safety findings from phase 3 clinical trials, with no new safety signals identified in patients with HAE aged ≥2 years treated with lanadelumab.^20^, ^25^, ^31^

Small sample sizes with low baseline attack rates can affect effectiveness outcomes, more so when patient outliers are reported in the dataset. This subanalysis has the advantage that the baseline (pre‐lanadelumab) rate of HAE attacks was moderately high (mean, 3.8 attacks per month) among adolescent patients who were new to lanadelumab, allowing scope for demonstration of substantial reductions in attack frequency on treatment. However, there are a number of limitations to this analysis. Confounding is a threat to the validity of findings obtained from observational studies, but this was mitigated by utilizing a self‐controlled design using regression analysis. Other limitations include the small sample size due to the rarity of HAE, which can be further affected by outliers, potential bias resulting from the inclusion of established lanadelumab patients, and possible misclassification of lanadelumab exposure and outcomes data due to data capture and recall bias.

In conclusion, these real‐world data from the EMPOWER and ENABLE studies suggest that lanadelumab provides effective LTP of HAE among adolescent patients in the real‐world clinical setting, consistent with results from studies in mixed adult/adolescent populations, supporting lanadelumab as a first‐line treatment option for management of HAE. No new safety signals were identified in this age group.

## AUTHOR CONTRIBUTIONS

**Raffi Tachdjian:** Investigation; writing – original draft; writing – review and editing; visualization. **Aleena Banerji:** Investigation; writing – original draft; writing – review and editing; visualization. **Paula J. Busse:** Investigation; writing – original draft; writing – review and editing; visualization. **Nancy Agmon‐Levin:** Investigation; writing – original draft; writing – review and editing; visualization. **John Anderson:** Investigation; writing – original draft; writing – review and editing; visualization. **Mauro Cancian:** Investigation; writing – original draft; writing – review and editing; visualization. **Giuseppe Spadaro:** Investigation; writing – original draft; writing – review and editing; visualization. **Carmen Enciu:** Conceptualization; methodology; investigation; writing – original draft; writing – review and editing; visualization. **Daniel Nova Estepan:** Conceptualization; methodology; software; formal analysis; validation; writing – original draft; writing – review and editing; funding acquisition; supervision; visualization; data curation; project administration; resources. **Natalie Khutoryansky:** Methodology; validation; writing – original draft; writing – review and editing; visualization; data curation. **Siddharth Jain:** Conceptualization; methodology; writing – original draft; writing – review and editing; funding acquisition; supervision; visualization; data curation; project administration; resources. **Andreas Recke:** Investigation; writing – original draft; writing – review and editing; visualization.

## FUNDING INFORMATION

The studies and this analysis were sponsored by Takeda Development Center Americas, Inc., Lexington, MA, USA.

## CONFLICT OF INTEREST STATEMENT

RT has received research/consulting fees from Astria, BioCryst, CSL Behring, Intellia, Ionis, KalVista, Pharming, Pharvaris, and Takeda and speaking honoraria from BioCryst, CSL Behring, Pharming, and Takeda; and served on advisory boards for BioCryst, CSL Behring, Ionis, KalVista, Pharming, and Takeda. AB has received institutional research/study support from Astria, Ionis, and Takeda; and/or honoraria for consulting from ADARx, Astria, BioCryst, CSL Behring, Intellia, KalVista, and Takeda. PJB has received research support and served on advisory boards for BioCryst, CSL Behring, and Takeda; and is a consultant for CVS Pharmacy, Medscape, Novartis, and Regeneron. NA‐L has no conflicts of interest to disclose. JA is a speaker bureau member for BioCryst, CSL Behring, Pharming, and Takeda; has received consulting fees from and is a clinical trial investigator for BioCryst, BioMarin, CSL Behring, KalVista, Pharming, Pharvaris, and Takeda; and has received consulting fees from Cycle Pharma. MC reports personal fees from BioCryst, CSL Behring, KalVista, Pharvaris, and Takeda. GS has received speaker/consultancy fees from AstraZeneca, Chiesi Farmaceutici, CSL Behring, GlaxoSmithKline, Sanofi, and Takeda. CE, DNE, NK, and SJ are employees of Takeda Development Center Americas, Inc. and hold stock options in Takeda Pharmaceutical Company Limited. AR has received research grants from Deutsche Forschungsgemeinschaft and EUROIMMUN; travel and conference support from BioCryst, Novartis, Pharming, Stallergenes Greer, and Takeda; speaker honoraria from Bencard, BioCryst, CSL Behring, EUROIMMUN, Novartis, and Takeda; and served as a consultant or participated in advisory boards for BioCryst, CSL Behring, Novartis, Swedish Orphan Biovitrum, and Takeda.

## Data Availability

The datasets, including the redacted study protocol, redacted statistical analysis plan, and individual participants' data supporting the results reported in this article, will be made available within 3 months from the initial request to researchers who provide a methodologically sound proposal. The data will be provided after its de‐identification, in compliance with applicable privacy laws, data protection, and requirements for consent and anonymization.
